# Few‐shot CBCT‐based synthetic CT generation with denoising diffusion probabilistic model

**DOI:** 10.1002/mp.70126

**Published:** 2025-11-13

**Authors:** Ping Lin Yeap, Xin Du, Meng Zhou, Andrew Hoole, Gillian C. Barnett, Rajesh Jena

**Affiliations:** ^1^ Department of Oncology University of Cambridge Cambridge UK; ^2^ Department of Physics University of Cambridge Cambridge UK; ^3^ Department of Medical Physics, Addenbrooke's Hospital Cambridge University Hospitals NHS Foundation Trust Cambridge UK; ^4^ Department of Oncology, Addenbrooke's Hospital Cambridge University Hospitals NHS Foundation Trust Cambridge UK; ^5^ Department of Biomedical Engineering The Chinese University of Hong Kong Hong Kong China

**Keywords:** adaptive radiotherapy, cone‐beam CT, diffusion model, synthetic CT generation

## Abstract

**Background:**

In adaptive cancer radiotherapy, treatment plans were periodically re‐optimised according to anatomical variations, with the goal of maintaining or improving clinical outcomes. This required high‐quality in‐room imaging for daily monitoring, tumour/organ segmentation and dose evaluation. However, in‐room cone‐beam computed tomography (CBCT) images suffered from inaccurate Hounsfield Unit (HU) values and poor image quality due to higher artifact presence and lower signal‐to‐noise ratio. While CBCT‐to‐CT generation models have shown promising results, many conventional networks require large datasets for training.

**Purpose:**

To address this, we proposed a denoising diffusion probabilistic model (DDPM) with a modified sampling process to enable few‐shot synthetic CT (sCT) generation from CBCT images.

**Methods:**

We used a denoising diffusion probabilistic model trained on rigidly registered CT‐CBCT image pairs in our approach, without requiring deformable registrations. Our model was trained on 25 patients from a head‐and‐neck cancer cohort at our institution, and validated and tested on separate sets of seven and eight patients. Our sampling method leveraged channel‐ and noised‐conditioning, demonstrating high‐quality sCT generation with limited training data. By adding noise to guiding CBCT images during sampling, we exploited the convergence of CBCT and planning CT (pCT) representations in latent space. This allowed sampling from a noisy representation of the data, achieving sCTs with high fidelity.

**Results:**

Evaluation on a head‐and‐neck (H&N) cancer dataset showed that sCTs outperformed CBCTs, reducing masked mean absolute error (MAE) from 131 ± 17 HU to 49 ± 7 HU, and improving peak signal‐to‐noise ratio (PSNR) from 20.0 ± 0.9 dB to 22.9 ± 1.1 dB and normalised cross‐correlation (NCC) from 0.93 ± 0.01 to 0.96 ± 0.01. We further evaluated the method's generalisability on phantoms and publicly available patient datasets. Without retraining, the model achieved moderate improvements over CBCT (e.g., masked MAE 87 ± 17 HU, PSNR 19.4 ± 1.7 dB, NCC 0.93 ± 0.04) on the external H&N dataset. However, after retraining on 27 in‐distribution patient cases, the model achieved comparable performance to our internal dataset, with MAE 48 ± 12 HU, PSNR 22.2 ± 2.5 dB, and NCC 0.95 ± 0.03. We also extended the model to the pelvis site, achieving similarly strong results (masked MAE 44 ± 9 HU, PSNR 21.9 ± 2.1 dB, NCC 0.96 ± 0.03), demonstrating the feasibility of our method across multiple anatomical sites. Visual inspections showed improved image quality, significantly reduced artifacts, and better anatomical preservation in sCTs. Clinician evaluations revealed a higher preference to use sCTs over pCTs for fractional evaluation.

**Conclusions:**

Our work offered a practical, data‐efficient and site‐robust solution for clinics to generate high‐fidelity sCTs, facilitating CBCT‐based dose evaluation and plan adaptation in adaptive radiotherapy workflows.

## 1 INTRODUCTION

In conventional radiotherapy, treatment was planned on an initial computed tomography (CT) scan taken during simulation and delivered in fractions over multiple weeks. Throughout the treatment course, inter‐fractional (due to weight gain or loss, organ displacements etc.) and intra‐fractional (due to breathing, bladder filling etc.) anatomical changes might occur, leading to discrepancies between planned and delivered dose.^1^, ^2^, ^3^ Adaptive radiotherapy aimed to re‐optimise the treatment plan based on periodic monitoring of anatomical variations, thereby maintaining or improving clinical outcomes.^4^, ^5^

To enable online adaptive replanning, high‐quality in‐room imaging was critical.^6^, ^7^ In image‐guided radiotherapy, most linear accelerators utilised on‐board cone‐beam computed tomography (CBCT) imaging for daily or weekly patient setup.^8^, ^9^ However, unlike fan‐beam CT images which were acquired slice‐by‐slice, CBCT images suffered from inaccurate Hounsfield Unit (HU) values and poorer image quality due to higher artifact presence and lower signal‐to‐noise ratio.^10^ This limited tumour and organ segmentation, dose calculations on CBCT images and hence CBCT‐based adaptive replanning.

In clinical workflows, artifacts could be manually corrected by overriding HU values at affected regions.^11^ Commercial treatment planning systems also offered various methods to enhance the HU calibration in CBCT images,^12^, ^13^ such as HU‐to‐electron density correction curves^14^ and deformable image registration (DIR) which deformed planning CT (pCT) images to daily CBCT images by finding an optimal geometric transformation between them.^15^, ^16^, ^17^ However, DIR might result in non‐physical or erroneous deformations, and the quality was often dependent on the operator's experience due to the number of adjustable hyperparameters.

Deep learning methods had shown increasingly promising results in generating synthetic CT (sCT) images to address the limitations in CBCT image quality and HU value accuracy.^18^, ^19^, ^20^ Chen et al. utilised a deep U‐Net architecture with a bottleneck layer that captured the most critical features while preserving spatial information, achieving low mean absolute error (MAE) between the sCT and reference CT. ^.^ ^21^ Other studies used cycle‐consistent generative adversarial networks (GANs) comprising a generator network to generate sCT images and a discriminator network to distinguish the sCT from the reference CT.^22^, ^23^, ^24^ While GANs had demonstrated improvements in image quality, artifact reduction, and soft tissue contrast, they were known to suffer from training instability and mode collapse.^25^ More recently, Peng et al. showed that diffusion‐based methods which used a multi‐step denoising network achieved superior sCT results compared to GANs.^26^ In this work, we focused on such physics‐inspired networks and proposed modifications that exploit the close underlying relationship between pCT and CBCT acquisition methods.

Learning models often required large datasets for training, which could be a limitation for individual clinics. While data federation methods had allowed localised training without sharing of patient data,^27^, ^28^ few studies had made use of large multi‐centre datasets for sCT generation. In this work, we proposed modifications to the training and sampling process in denoising diffusion probabilistic models (DDPMs)^29^, ^30^ and demonstrated their utility in generating high‐quality synthetic CT images with small patient training datasets. Our contributions were as follow:

1. We investigated different noise schedules and proposed a channel and noised‐conditioned sampling technique by leveraging the convergence of pCT and CBCT representations in latent space.
2. We showed that loss convergence was dependent on timesteps and proposed alternative non‐uniform timestep samplers.

We believed the methodology in this work would be relevant to centres which were planning to develop in‐house sCT generation models for CBCT‐based adaptive radiotherapy workflows.

## 2 METHODS

This study made use of the Addenbrookes Secure Therapeutic Radiology Image Dataset (ASTRID) which was approved by the NHS Research Ethics Committee (reference number 22/WM/0072).

### 2.1 Data and pre‐processing

The primary dataset comprised 40 retrospective head‐and‐neck (H&N) cancer patients treated with image‐guided volumetric modulated arc therapy (IG‐VMAT) at Cambridge University Hospitals (CUH) between 2016 and 2019. These patients received a minimum dose prescription of 60 Gy in 30 fractions. Immobilisation devices and CBCT guidance images were used for daily set‐up reproducibility. For each patient, the pCT and CBCT images acquired for the first fraction were used, as this ensured minimal anatomical changes due to radiotherapy treatment. The pCT and CBCT scans were rigidly registered to simulate couch shifts, giving a total of 1336 image pairs. To mirror clinical workflows and avoid introducing erroneous deformations, no DIR was performed. The mean duration between the pCT and CBCT scans was 18.2 days (range, 6–27 days). The 40 patients were split into train, validation, and test groups using a 25:8:7 ratio. The training set was curated to include patients with pCT and CBCT images that were most closely matched (i.e., minimal anatomical changes between the two scans) and with minimal metal artifacts in the pCTs. This deliberate selection was made to ensure reliable supervision for the model, especially given the sensitivity of generative models to inconsistencies in paired data. The remaining cases were randomly split into validation and test sets.

Next, we obtained a phantom dataset comprising two sets of CT and CBCT scans acquired from two rigid H&N phantoms constructed with materials simulating soft tissue and bone densities: ExacTrac Dynamic Cranial Verification Phantom (Brainlab, Germany) and Stereotactic End‐to‐End Verification Phantom (CIRS Inc., USA). As the phantom CT and CBCT scans were anatomically exact, they served as an additional dataset for model testing.

The pCT images for the CUH patients and phantoms were acquired on Aquilion LB (Toshiba Medical Systems, Japan) using 120 kVp, with a voxel size of 1.074 × 1.074 × 3 mm^3^. The CBCT images were acquired on Elekta XVI (Elekta AB, Sweden) with a voxel size of 1.0 × 1.0 × 3.0 mm^3^ for 24 patients and on Varian On‐Board Imager (Varian Medical Systems, USA) with a voxel size of 0.511 × 0.511 × 2 mm^3^ for the remaining 16 patients using 100–120 kVp.

Finally, to validate our model on an external dataset and demonstrate feasibility across different anatomical sites, we used H&N and pelvis patient data from the publicly available SynthRAD2023 dataset collected from 3 Dutch university medical centers.^31^ For each site, we curated a set of 27 patients (nine from each center) for training and an additional nine random patients for testing.

For pre‐processing, all scans were resampled to the same voxel size of 1.0 × 1.0 × 3.0 mm^3^ using linear interpolation. Each image was center‐cropped to 256 × 256 pixels for H&N and 512 × 512 pixels for pelvis, which were equivalent to the CBCT field‐of‐view (FOV). Pixel values were clipped to [−1000, 3000] and rescaled to [−1, 1].

### 2.2 Denoising diffusion probabilistic model

Diffusion is a Markovian process, which is described by a sequence of events where the probability of each event depends only on the state of the previous event. By introducing noise iteratively, we degraded the data, pushing it towards a simpler (Gaussian) distribution. A neural network could then learn to reverse this process akin to “de‐noising”, starting from the simple distribution and gradually converging towards the desired complex real‐world data distribution through iterative refinements, hence restoring the data. It should be noted that the Gaussian noise schedules for degrading the data were not related to the inherent quantum noise in imaging systems.

The data at each step in the forward diffusion process, *q*, can be defined by Equation (1):

(1) $$q\;\left(x_{t}|x_{t-1}\right)=N\left(x_{t};\sqrt{1-\beta_{t}}x_{t-1},\;\beta_{t}I\right)$$

where the amount of Gaussian noise added at each timestep, *t*, is governed by the noise schedule $\beta_{t}$. In this work, we explored 3 different noise schedules^32^ ^—^linear, cosine, and sigmoid—with $\beta_{t}$ ranging from 0.0001 to 0.02 over a total of *T* = 1000 timesteps.

To generate a training sample at *T*, noise could be added iteratively using Equation (2), which was computationally slow:

(2) $$q\;\left(x_{1:T}|x_{0}\right)=\prod_{t=1}^{T}q\left(x_{t}|x_{t-1}\right)$$

By letting $\alpha_{t}=1-\beta_{t}$ and ${\bar{\alpha }}_{t}=\prod_{i=1}^{t}\alpha_{i}$, the noise could be conveniently computed at any step *t* in one step:

(3) $$q\;\left(x_{t}|x_{0}\right)=N\left(x_{t};\sqrt{{\bar{\alpha }}_{t}}x_{0},\;\left(1-{\bar{\alpha }}_{t}\right)I\right)$$

(4) $$x_{t}=\sqrt{{\bar{\alpha }}_{t}}\;x_{0}+\sqrt{1-{\bar{\alpha }}_{t}}\varepsilon$$

where ε was from N(0,1).

The reverse diffusion process aimed to gradually restore an image from pure noise, by training a neural network to predict the entire noise that was added at a given timestep. The predicted noise was scaled based on the schedule and subtracted from the input image. This was repeated until *t* = 0. The process was described by Equation (5), where θ were the parameters of the neural network updated by gradient descent.

(5) $$p_{\theta }\left(x_{0:T}\right)=p\left(x_{T}\right)\prod_{t=1}^{T}p_{\theta }\left(x_{t-1}|x_{t}\right)$$

where

(6) $$p_{\theta }\;\left(x_{t-1}|x_{t}\right)=N\left(x_{t-1};\mu_{\theta }\left(x_{t},t\right),\;\sum_{\theta }\left(x_{t},t\right)\right)$$

In this work, we used a 2D U‐Net architecture^33^ with six down‐sampling and six up‐sampling blocks, each containing two ResNet layers. Most blocks were standard ResNet blocks, but one down‐sampling and one up‐sampling block included spatial self‐attention to enhance detail preservation. The network progressively increased the number of channels per block to capture more complex features, making it well‐suited for generating high‐quality images with both fine details and robust spatial structures. The schematic diagram of our U‐Net for each step of the training and sampling processes was illustrated in Figure 1.

FIGURE 1Training and sampling processes. (a) Training process showing the latent space representation (in blue) and pCT‐CBCT concatenation across the forward diffusion and reverse denoising processes. The U‐Net architecture with inputs and outputs are shown for one sampled timestep during denoising training. (b) Sampling process showing the denoising process beginning from a guiding CBCT image with partial noise added. The latent space representation (in blue and red) shows the noisy CBCT converging with the noisy CT in latent space, allowing for better CBCT‐guided sCT generation.

**Figure:**
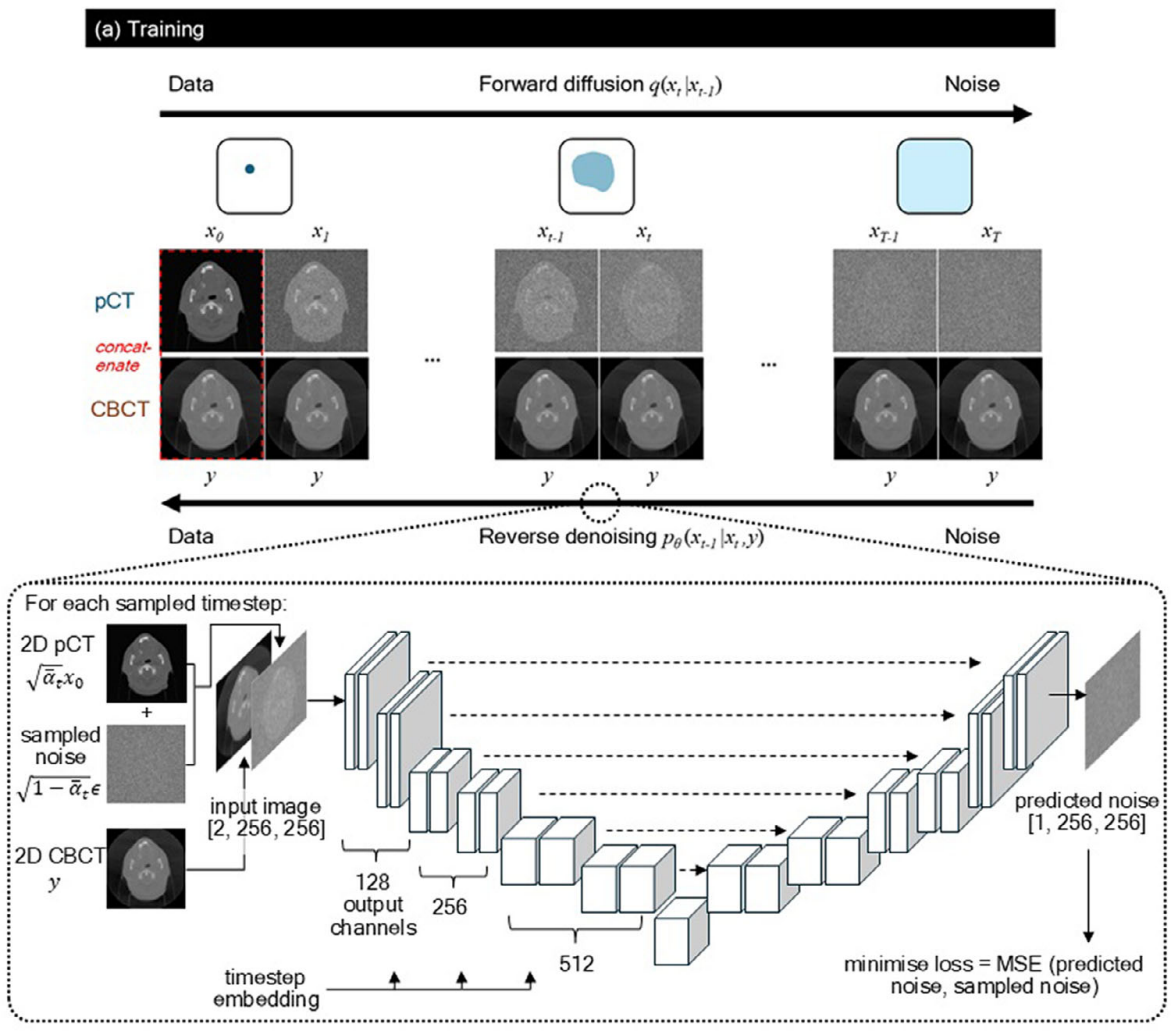

**Figure:**
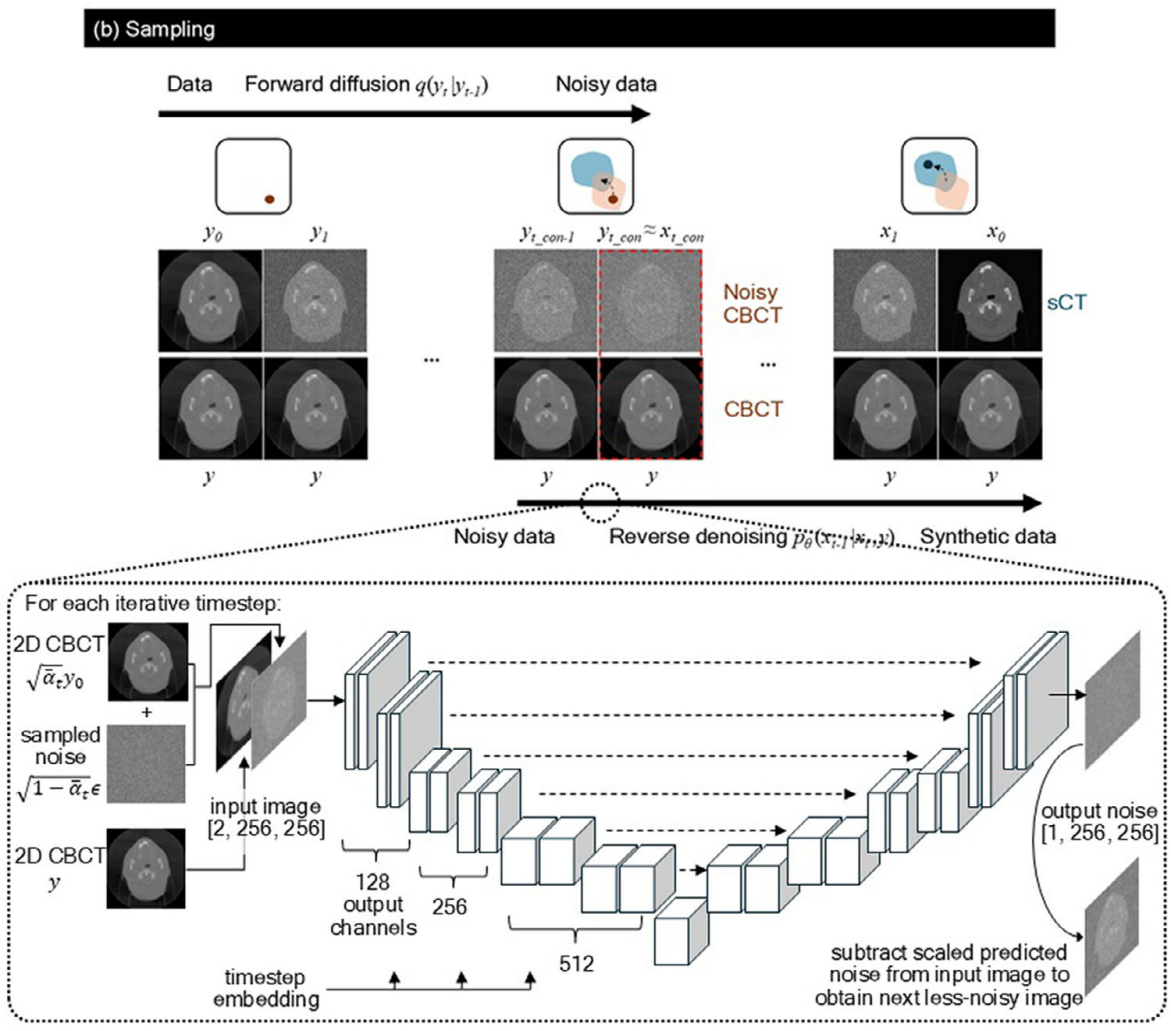

The choice of using six residual blocks in the U‐Net backbone was inspired by configurations used in prior work on diffusion models for image synthesis, which were shown to be effective. In particular, Ho et al.’s work on denoising diffusion probabilistic models^29^ used three resolution levels with two convolutional residual blocks per resolution level and self‐attention blocks at the 16 × 16 resolution between the convolutional blocks. Saliman et al.’s PixelCNN++ model consisted of six blocks of five ResNet layers.^34^ We also felt that using six residual blocks gave a balance between model expressiveness and computational efficiency, especially in the context of limited paired training data and the high‐dimensional nature of 3D medical images. This depth provided sufficient receptive field coverage to capture both local and global anatomical features, which was critical in CBCT‐to‐CT synthesis tasks.

### 2.3 Proposed channel and noised‐conditioned sampling

To enable image conditioning,^35^ the U‐Net was modified to accept inputs with two channels instead of one. During training, noise was added to the CT image in the forward process while the CBCT image remained noise‐free. The guiding CBCT image y was concatenated with the noisy CT sample $x_{t}$ along the channel dimension (“*channel‐conditioned*”). During sampling, the CBCT image y was similarly concatenated with each target image $x_{T:0}$, allowing the CBCT image to guide the denoising process at each timestep. The output of the U‐Net was a 1‐channel noise tensor, which was then removed from $x_{t}$ and passed to the next timestep. Training was performed on NVIDIA A100 80GB GPUs (Ampere architecture) on the Cambridge University high performance computing platform. Our network had about 110 M parameters. To ensure a standardised comparison, all experiments were trained for 12 h on one GPU node, which corresponded to 180 epochs for our primary training dataset.

**FIGURE 2:**
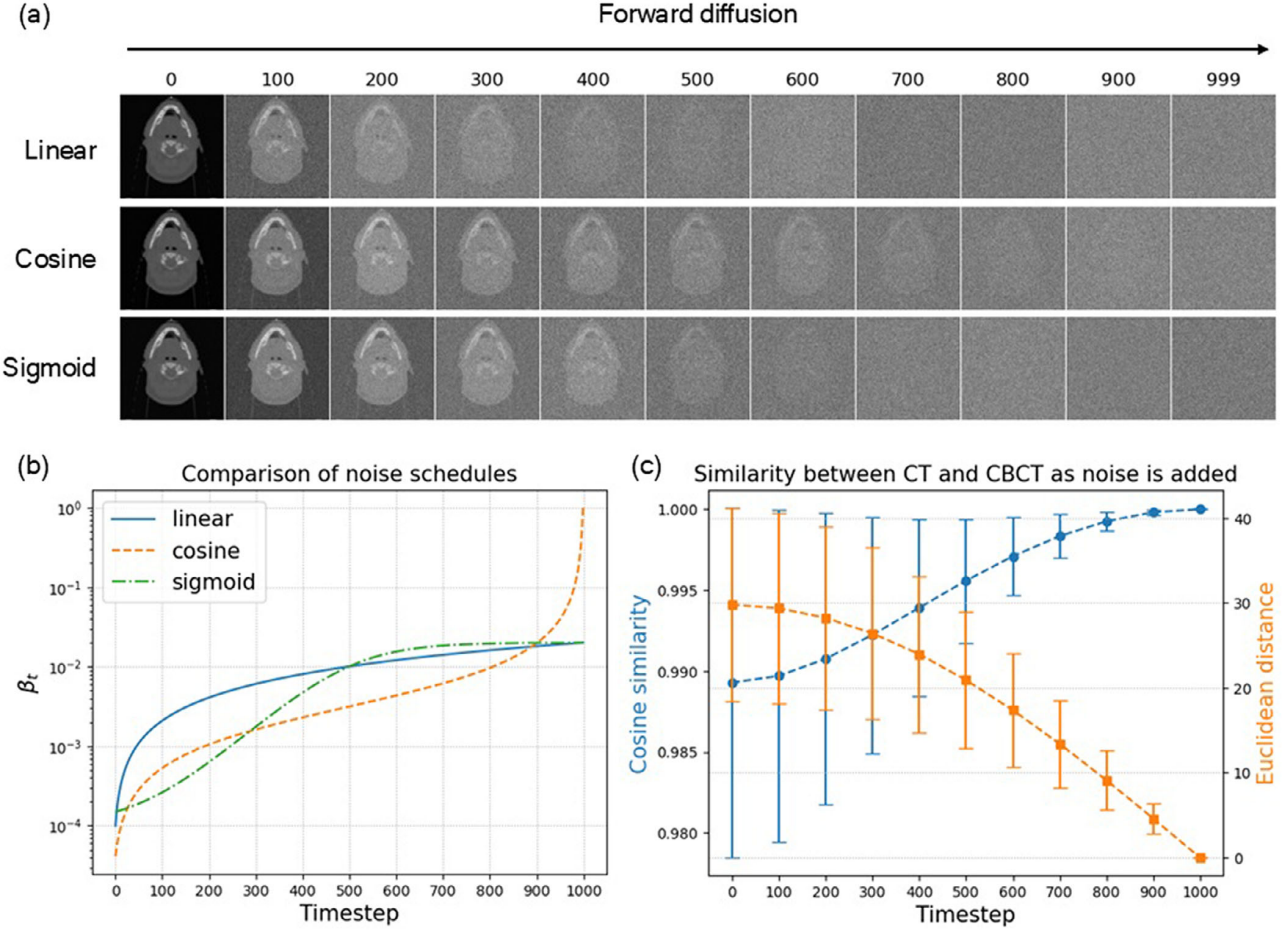
Noise schedules. (a) Noise added to pCT image during the forward diffusion process using linear, cosine, and sigmoid noise schedules. In this work, the forward diffusion process runs in the direction of increasing *t*. (b) Plot of *β_t_* (i.e., proportion of noise added to image) at each timestep for linear (blue solid), cosine (orange dashed), and sigmoid (green dotted) noise schedules. (c) Plot of cosine similarity (blue) and Euclidean distance (orange) between the pCT and CBCT images as noise was added at each timestep.

In conventional DDPM, the sampling process begins from $x_{T}$, which was sampled from pure Gaussian noise N(0,1). While the CBCT channel served as a guide for the sampling process, we introduced an additional conditioning mechanism in this work: instead of beginning the denoising process from pure noise, we added noise to the guiding CBCT using the same noise schedule up till a timestep $t_{con}$ to obtain $y_{t\_con}$, then begin denoising (“*noised‐conditioned”*). Since CT and CBCT images were both acquired using similar imaging modalities (i.e. measuring kilovoltage X‐ray attenuation in tissue), the intuition was that the noisy versions of them would converge in the latent space and have similar representations in the later timesteps of the diffusion process. Further, as seen from Equation (4), as ${\bar{\alpha }}_{t}$ decreased with increasing t, the signal term $\sqrt{{\bar{\alpha }}_{t}}x_{0}$ diminished over timesteps and the noise term $\sqrt{1-{\bar{\alpha }}_{t}}\varepsilon$ became dominant. $t_{con}$ became a tune‐able hyperparameter, and the challenge was therefore to find the optimal timestep which determined the amount of noise added: too late a timestep meant much of the meaningful anatomical information in the CBCT image got destroyed, while too early a timestep meant the neural network might not have sufficient iterations to improve the image quality. The training and *channel and noised‐conditioned* sampling processes were illustrated in Figure 1, while the noise schedules were illustrated in Figure 2.

Next, we investigated the impact of the choice of timestep sampler. Conventional DDPMs sampled timesteps from a uniform distribution across 0 to *T* during training. We studied the rate of convergence with different timesteps and explored the use of exponential and triangular distributions for timestep sampling. The algorithms of the original DDPM and our proposed modified DDPM were given in Algorithms 1 and 2.

Algorithms 1 and 2: Training and sampling processes of original DDPM (Algorithm 1) and our proposed modified DDPM (Algorithm 2).

**Table:** 

**Algorithm 1.1** Training	**Algorithm 1.2** Sampling
1: **repeat**	1: $x_{T}\;∼\;N\left(0,I\right)$
2: $x_{0}\;∼\;q\left(x_{0}\right)$	2: **for** t=T,…,1 **do**
3: t∼Uniform({1,…,T})	3: z∼N(0,I) if t>1, else z=0
4: ε∼N(0,I)	4: $x_{t-1}=$
5: $x_{t}=\sqrt{{\bar{\alpha }}_{t}}\;x_{0}+\sqrt{1-{\bar{\alpha }}_{t}}\varepsilon$	$\frac{1}{\sqrt{{\bar{\alpha }}_{t}}}\left(x_{t}-\frac{1-\alpha_{t}}{\sqrt{1-{\bar{\alpha }}_{t}}}\varepsilon_{\theta }\left(x_{t},t\right)\right)+\sigma_{t}z$
6: Take a gradient step on	5: **end for**
$\nabla_{\theta }{||\varepsilon -\varepsilon_{\theta }\left(x_{t},t\right)||}^{2}$	6: **return** $x_{0}$
7: **until** converged

**Table:** 

**Algorithm 2.1** Training	**Algorithm 2.2** Sampling
1: **repeat**	1: ε∼N(0,I)
2: $\left(x_{0},y_{0}\right)\;∼\;q\left(x_{0},y_{0}\right)$	2: $x_{t\_con}=\sqrt{{\bar{\alpha }}_{t\_con}}\;y_{0}+\sqrt{1-{\bar{\alpha }}_{t\_con}}\varepsilon$
3: t∼Sampler({1,…,T})	3: **for** t=t_con,…,1 **do**
4: ε∼N(0,I)	4: z∼N(0,I) if t>1, else z=0
5: $x_{t}=\sqrt{{\bar{\alpha }}_{t}}\;x_{0}+\sqrt{1-{\bar{\alpha }}_{t}}\varepsilon$	5: $x_{t-1}=$
6: Take a gradient step on	$\frac{1}{\sqrt{{\bar{\alpha }}_{t}}}\left(x_{t}-\frac{1-\alpha_{t}}{\sqrt{1-{\bar{\alpha }}_{t}}}\varepsilon_{\theta }\left(x_{t},y_{0},t\right)\right)+\sigma_{t}z$
$\nabla_{\theta }{||\varepsilon -\varepsilon_{\theta }\left(x_{t},y_{0},t\right)||}^{2}$	6: **end for**
7: **until** converged	7: **return** $x_{0}$

Finally, we performed a sensitivity analysis to evaluate the effect of training data size on model performance. Specifically, we trained separate instances of the model using progressively fewer patient scans, with training set sizes ranging from 25 to 5 patients. All other training parameters, including model architecture and number of epochs, were held constant across experiments to isolate the effect of training size. This analysis aimed to understand the robustness of our proposed approach under data‐limited conditions.

### 2.4 Evaluation

In this study, the sCTs were evaluated across three axes: image quality, alignment to conditioning CBCTs, and clinicians’ feedback. To assess the quality of the sCT images, we calculated the mean absolute error (MAE), peak signal‐to‐noise ratio (PSNR), and normalized cross‐correlation (NCC) metrics as given in Equations (7), (8), and (9) between sCT and the registered pCT images, and compared them against the metrics between the corresponding CBCT and pCT images. However, as the CBCT images had a smaller FOV than the pCT images, we created circular image masks based on the small CBCT FOV and calculated metrics on the masked images.

Since the pCT and CBCT images in the validation and test sets were only rigidly registered, there could be residual set‐up errors and anatomical differences (e.g. due to weight loss or tumour growth between simulation and treatment). As a result, the quantitative metrics might be unable to disentangle these errors from the sCT generation errors. To assess alignment of the sCT with the conditioning CBCT scans, we therefore looked at HU difference maps between the image pairs. In addition, we evaluated the model on phantom scans (which were rigid and would not have anatomical differences) and used a smaller circular mask to exclude set‐up errors in the phantom headrest.

Finally, it was equally important to obtain qualitative feedback from clinicians on the quality of the image and their propensity to choose sCT over pCT images for dose evaluation. To this end, we presented 20 random pCT‐CBCT‐sCT image sets to 3 experienced clinicians (2 radiation oncologists and 1 medical physicist) from CUH. We first investigated the “quality score” where clinicians compared the labelled sCT, CBCT and pCT images and scored the quality of the sCT images on a 5‐point Likert scale (1 for poor and unusable quality; 5 for excellent and CT‐equivalent quality). We also investigated the “preference rate” which was given by the rate clinicians picked sCTs over pCTs as their preferred image for fractional segmentation and dose evaluation.

## 3 RESULTS

### 3.1 CBCT‐guided conditioning mechanisms

Table 1 showed the masked MAE, PSNR and NCC values for the linear, cosine, and sigmoid noise schedules at different values of *t_con_* on the primary validation set. With only channel‐conditioning, where sampling began from pure Gaussian noise (i.e., *t_con_* = *T* = 1000), the model generated worse sCTs than the CBCTs, with the worst result produced by the cosine schedule (sCT MAE = 1003 ± 376 HU vs. CBCT MAE = 130 ± 16). The metrics improved drastically when noised‐conditioned sampling was added (i.e., sampling from a noised CBCT with *t_con_* < 1000, instead of pure noise). The rate of improvement was highest for a cosine schedule where MAE dropped sharply to 49 ± 7 HU with *t_con_* = 900, while the improvement rates were more gradual for the linear and sigmoid schedules. With channel and noised‐conditioned sampling, the best results for sCT on the validation set were achieved with a cosine noise schedule and *t_con_* = 800, with MAE = 48 ± 5 HU, PSNR = 26.5 ± 1.1 dB, and NCC = 0.97 ± 0.01, compared to CBCT with MAE = 130 ± 16 HU, PSNR = 23.7 ± 1.5 dB, and NCC = 0.94 ± 0.02.

**TABLE 1:** Evaluation metrics of masked mean absolute error (MAE), peak signal‐to‐noise ratio (PSNR), and normalized cross‐correlation (NCC) calculated on sCT and CBCT images relative to pCT images using linear, cosine, and sigmoid noise schedules.

		sCT			
	*t_con_*	Linear	Cosine	Sigmoid	CBCT
Masked MAE/HU (↓)	1000	436 ± 261	1003 ± 376	478 ± 274	130 ± 16
	900	195 ± 136	49 ± 7	205 ± 135	
	800	87 ± 53	**48 ± 5**	80 ± 48	
	700	54 ± 16	49 ± 5	50 ± 10	
	600	**48 ± 6**	50 ± 5	**48 ± 5**	
	500	**48 ± 5**	50 ± 6	49 ± 5	
	400	49 ± 5	51 ± 6	50 ± 6	
Masked PSNR/dB (↑)	1000	14.2 ± 5.1	7.5 ± 3.4	13.3 ± 5.1	23.7 ± 1.5
	900	20.4 ± 5.0	**26.5 ± 1.1**	20.0 ± 4.9	
	800	24.3 ± 3.1	**26.5 ± 1.1**	24.7 ± 3.0	
	700	26.0 ± 1.7	26.4 ± 1.2	26.3 ± 1.5	
	600	26.4 ± 1.4	26.4 ± 1.2	26.4 ± 1.3	
	500	26.4 ± 1.3	26.4 ± 1.2	26.4 ± 1.3	
	400	26.3 ± 1.3	26.3 ± 1.3	26.3 ± 1.3	
Masked NCC (↑)	1000	0.63 ± 0.23	0.24 ± 0.24	0.66 ± 0.18	0.94 ± 0.02
	900	0.87 ± 0.11	**0.97 ± 0.01**	0.86 ± 0.10	
	800	0.95 ± 0.03	**0.97 ± 0.01**	0.95 ± 0.03	
	700	0.96 ± 0.01	0.96 ± 0.01	0.96 ± 0.01	
	600	0.96 ± 0.01	0.96 ± 0.01	0.96 ± 0.01	
	500	0.96 ± 0.01	0.96 ± 0.01	0.96 ± 0.01	
	400	0.96 ± 0.01	0.96 ± 0.01	0.96 ± 0.01	

*Note*: Arrows (↓ or ↑) indicate if metrics should be minimised or maximised. Best‐performing values are in **bold**. If *t_con_* = 1000, the sCT was sampled from pure Gaussian noise.

**FIGURE 3:**
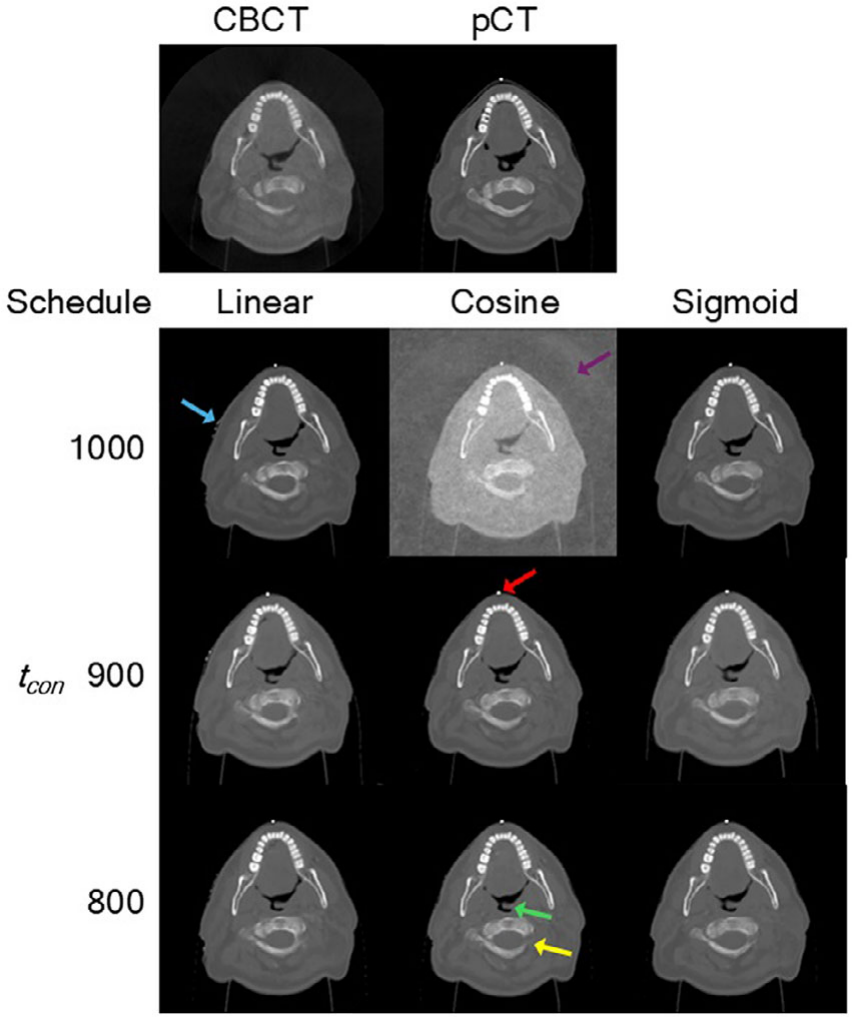
Synthetic CT images generated from different noise schedules and *t_cos_*. Top 2 images show the CBCT and pCT, while below 9 images show the different generated sCTs. Purple arrow: Poor generation quality with cosine schedule and *t_cos_* = 1000 due to inaccurate air CT values in guiding CBCT. Blue arrow: Hallucination of mask with linear schedule. Red arrow: Hallucination of set‐up BB ball in all generated images. Green arrow: Uvula was different in pCT image but followed CBCT anatomy in generated images. Yellow arrow: Best spine generation with cosine schedule and *t_cos_* = 800.

Figure 3 shows the sCT images generated from one CBCT image example. The sCTs generated with added noised‐conditioning appeared visibly better than the CBCTs, with fewer noise and streaking artefacts. In addition, certain anatomical regions in the CBCT image, such as the uvula and the body contours, had changed since the acquisition of the pCT image but were preserved in the sCT images. However, some hallucinations were also observed, such as the set‐up ball bearing that appeared in generated sCTs but not the guiding CBCT, as well as the body mask that appeared in sCTs generated with a linear schedule. The best sCT images were generated with the cosine schedule and preserved the spinal shape on the CBCT. On the other hand, the sCT generated with a cosine schedule and *t_con_* = 1000 appeared noisiest, with the original CBCT FOV visible in the background.

### 3.2 Timestep sampler

Conventionally, the DDPM used a uniform timestep sampling distribution, where the timestep *t* was sampled uniformly from [0, *T*] during the training process. From Figure S‐1a, it was observed that the loss aggregated over all batches quickly converged after a few epochs and remained steadily low, even though the quality of the generated images was continually improving. By investigating the loss curves for different timestep bands in Figure S‐1c, we found that the loss curves for the lower timestep bands, where the image had been mostly de‐noised and the finer details were being formed, converged slower than the loss curves for the higher timestep bands, where the image was still mostly noisy and the coarse structures were being formed.

As it seemed harder for the model to learn to de‐noise at lower timesteps, we explored two other timestep distributions, exponential and triangular, that sampled more frequently towards the lower timesteps, as shown in Figure S‐1b. From Figure S‐1d, we found that the loss curves for the lower bands using these distributions converged faster than those with uniform distribution, although they still converged slower than the higher bands. In addition, the loss curves at the higher bands performed comparably well across all distributions.

Since the noised‐conditioned approach was designed to begin sampling from *t_con_* and therefore did not require de‐noising at the higher timesteps, it also seemed logical to use timestep distributions that were weighted towards the lower timesteps. However, the models trained with the exponential and triangular distributions did not achieve better sCT results than the linear distribution, with MAE of 48 ± 6 HU, 49 ± 5 HU, and 48 ± 5 HU respectively.

### 3.3 Visual quality inspection

**FIGURE 4:**
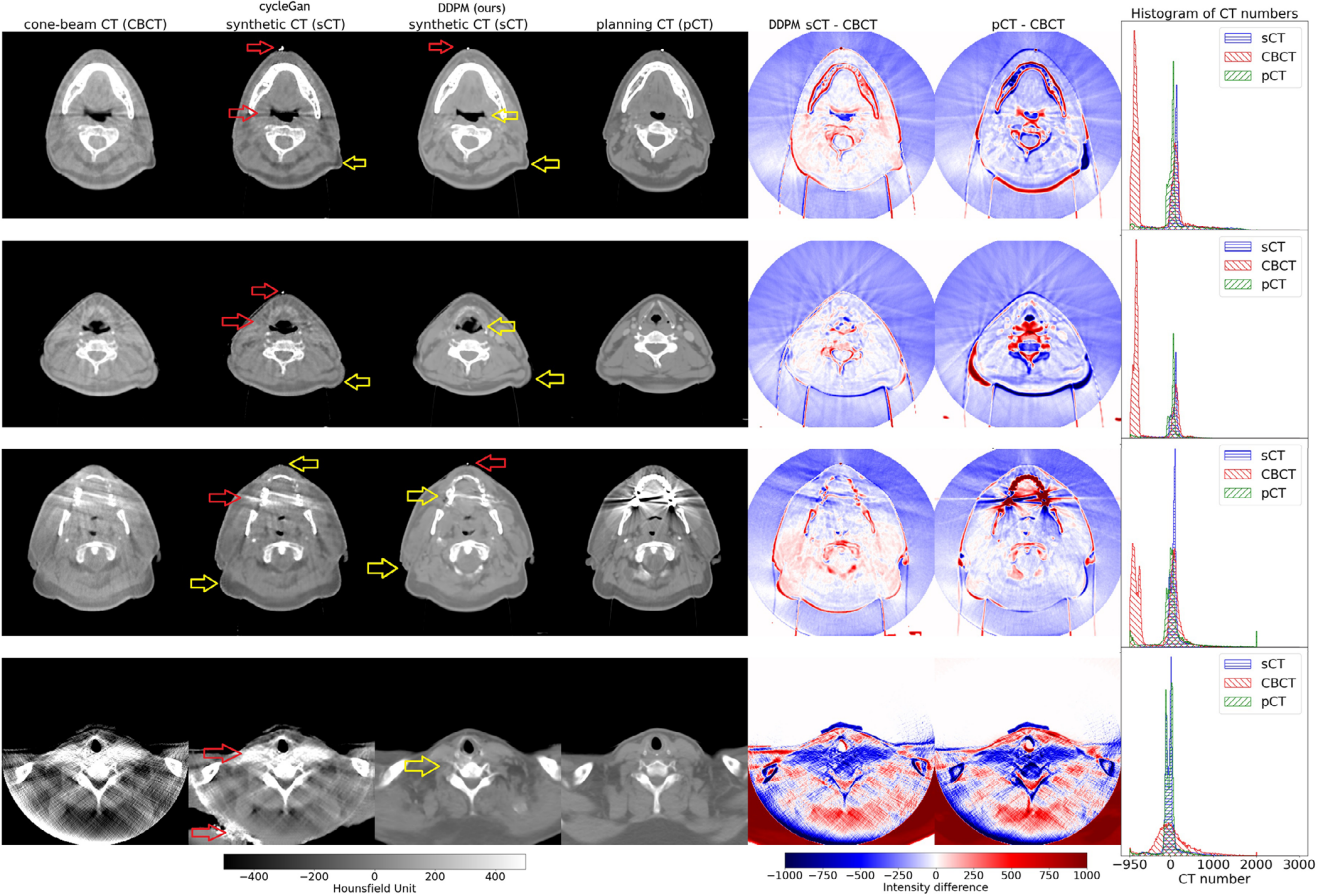
Samples of our DDPM‐generated sCT with the guiding CBCT and corresponding pCT images, HU difference maps, and histogram of CT numbers for CUH patient test images. Samples of cycleGAN‐generated sCTs were also displayed for comparison. Improved sCT features were annotated with yellow arrows, while poor‐performing sCT features were annotated with red arrows. Note: histogram was truncated to −950 HU at the lower limit to exclude air, which would otherwise overwhelm other HU values.

Using channel and noised‐conditioned sampling with a cosine noise schedule, uniform timestep distribution, and *t_con_* = 800, the masked MAE, PSNR and NCC values on the sCT and CBCT images for the CUH patient test, phantom, and SynthRAD datasets could be found in Table 2. Evaluation on the CUH test dataset showed that the DDPM‐generated sCTs outperformed the CBCTs, reducing the masked MAE from 131 ± 17 HU to 49 ± 7 HU, and improving the PSNR from 20.0 ± 0.9 dB to 22.9 ± 1.1 dB and the NCC from 0.93 ± 0.01 to 0.96 ± 0.01. To better evaluate the performance of our method, we also trained a cycleGAN for the same number of epochs using the recommended architecture in the original implementation,^36^ which included 9 residual blocks in the generator for 256 × 256 resolutions, a cycle‐consistent weighting factor of *λ* = 10, and an identity mapping loss of 0.5*λ*. We used cycleGAN for this comparison because our pCT‐CBCT image pairs were not perfectly aligned due to anatomical deformations and FOV differences, making the pix2pix model^37^ unsuitable for this task. The cycleGAN achieved metrics comparable to our DDPM approach, with masked MAE of 50 ± 8 HU, PSNR of 22.8 ± 1.2 dB, and NCC of 0.96 ± 0.01. However, despite the similar quantitative results, visual inspection (Figure 4) revealed that the cycleGAN‐generated sCTs did not show perceptible improvements in image quality over the CBCTs.

**TABLE 2:** Evaluation metrics of masked mean absolute error (MAE), peak signal‐to‐noise ratio (PSNR), and normalized cross‐correlation (NCC) calculated on our DDPM‐generated sCT and CBCT images for the primary CUH patient, phantom, and SynthRAD test sets using optimised hyperparameters. For the CUH test set, metrics for cycleGAN‐generated sCT were also given.

	CUH H&N (*n *= 7)			Phantom H&N (*n *= 2)		SynthRAD H&N (*n *= 9)			SynthRAD pelvis (*n *= 9)	
	cycleGAN sCT	DDPM sCT (ours)	CBCT	sCT	CBCT	sCT (no retrain)	sCT (retrained)	CBCT	sCT	CBCT
Masked MAE / HU (↓)	50 ± 8	49 ± 7	131 ± 17	31 ± 2	43 ± 2	87 ± 17	48 ± 12	102 ± 21	39 ± 7	106 ± 15
Masked PSNR / dB (↑)	22.8 ± 1.2	22.9 ± 1.1	20.0 ± 0.9	28.8 ± 1.8	27.1 ± 1.8	19.4 ± 1.7	22.2 ± 2.5	19.6 ± 1.4	23.6 ± 1.3	19.3 ± 1.2
Masked NCC (↑)	0.96 ± 0.01	0.96 ± 0.01	0.93 ± 0.02	0.992 ± 0.003	0.992 ± 0.002	0.93 ± 0.04	0.95 ± 0.03	0.92 ± 0.03	0.96 ± 0.01	0.94 ± 0.02

*Note*: *n* refers to the number of 3D scans in the dataset.

In the first two examples of Figure 4, our DDPM model successfully preserved the oesophagus shape in the CBCT, despite anatomical differences with the pCT, demonstrating its ability to reflect daily anatomical variations. Additionally, in the first three examples, the sCTs preserved body contour changes (likely due to patient weight gain or loss) between the CBCT and pCT, reinforcing its ability to adapt to inter‐fraction anatomical changes. In the third example, the sCT also effectively reduced metal streaking artifacts from dental implants. By contrast, while the cycleGAN also reproduced the overall body contours of the CBCT, they failed to reduce any streaking artifacts in the CBCTs in all 4 examples.

The fourth example showed a slice in the thorax region, which gave severe image degradation in the CBCT due to greater X‐ray attenuation. However, our DDPM‐generated sCT was able to recover the underlying image quality, though regions outside the CBCT FOV were extrapolated and could be inaccurate. By contrast, the cycleGAN generated unphysical body contours and severe uncorrected artifacts.

One limitation of both models was observed in the first three examples, where they hallucinated setup ball bearings that were not present in the input CBCTs, suggesting potential issues with overfitting to training data or reliance on prior patterns. In all 5 examples, the histogram of CT numbers for sCT closely resembled the pCT compared to the CBCT.

### 3.4 Clinician evaluation

The mean quality score of the sCT images given by the 3 clinicians were 3.45, 3.8 and 4.8 out of 5. The preference rate of the sCT images for dose evaluation were 65%, 70% and 85%. In terms of qualitative feedback, clinicians preferred the sCT because of reduced artifacts and body shape preservation from the CBCT. In cases where clinicians preferred the pCT, it was primarily due to better contrast between soft tissues, clearer blood vessel visualisation, and/or a complete FOV of the couch structure on the pCT images. In cases where clinicians did not spot noticeable differences between the sCT and pCT, they preferred pCT images as a safety precaution.

### 3.5 External validation

As shown in Table 2, applying the model trained on the primary CUH dataset directly to the external SynthRAD H&N dataset resulted in a masked MAE of 87 ± 17 HU, PSNR of 19.4 ± 1.7 dB, and NCC of 0.93 ± 0.04 for the generated sCT images, compared with 102 ± 21 HU, 19.6 ± 1.4 dB, and 0.92 ± 0.03 for the input CBCT. After retraining the network from scratch using the 27 patients from the SynthRAD training set, performance improved to a masked MAE of 48 ± 12 HU, PSNR of 22.2 ± 2.5 dB, and NCC of 0.95 ± 0.03. These results were consistent with the performance observed on the primary CUH test set. Figure S‐2a showed four examples of sCT generation using the SynthRAD H&N dataset. In the first two examples, despite observable setup differences between the pCT and CBCT, the generated sCTs preserved the anatomy and body contour seen in the CBCT while also improving image quality. In the third example, streaking artifacts present in the CBCT were not significantly reduced in the resulting sCT. In the fourth example, a treatment bolus that was present during the CBCT acquisition was also reproduced in the sCT.

Figure S‐2b showed 4 examples from the SynthRAD pelvis images. The CBCT images in the pelvis region were visibly of lower quality than their corresponding pCT images, with inaccurate HU values and frequent artifacts. In the first two examples, streaking artifacts caused by metallic implants in the femora were clearly visible in the CBCT and pCT images but were noticeably reduced in the generated sCT. Remarkably, the sCT exhibited fewer artifacts than even the pCT. However, in the second example, this artifact suppression led to over‐correction, resulting in the absence of the left femoral head in the sCT. In the third example, the patient's body habitus was smaller in the CBCT than in the pCT (likely due to weight loss) and this change was accurately reflected in the sCT, preserving the external body contour. Additionally, a pocket of bowel gas seen in the CBCT but not in the pCT was successfully reproduced in the sCT. However, not all bowel gas pockets were consistently generated, as demonstrated in the fourth example. Additionally, in both the third and fourth examples, the sCT included a small couch structure not present in the CBCT but seen in simulation CTs. This likely occurred because the model was trained on pCT images where such structures were often present. Across all four examples, the intensity histograms of the sCTs closely matched those of the pCTs compared to the CBCTs, indicating improved HU fidelity. Correspondingly, all quantitative evaluation metrics including MAE, PSNR, and NCC were significantly improved in the sCT compared to the CBCT, as summarised in Table 2.

In the phantom examples from Figure S‐2c, the histogram of CT numbers in the sCT images did not follow as closely to the pCT images. This was mostly attributed to a non‐uniform generation of tissue HU values, as well as set‐up errors in the phantom headrest.

### 3.6 Sensitivity analysis

As shown in Figure S‐3a, the evaluation metrics degraded only marginally with reduced training data. Even when trained with just 5 patient scans, the model achieved strong performance, with masked MAE, PSNR, and NCC values of 52 ± 7 HU, 22.7 ± 1.0 dB, and 0.96 ± 0.01, respectively. However, the loss curve in Figure S‐3b revealed signs of overfitting, as models trained with fewer scans tended to converge more quickly. Figure S‐3c illustrated representative examples of generated sCT images. While the models preserved the overall body contour from the CBCT, soft tissue contrast visibly diminished as the size of the training dataset decreased.

## 4 DISCUSSION

In this work, we showed that, with the appropriate sampling method, DDPMs could be trained on small datasets and generate high fidelity sCT images based on CBCT images. While both the DDPM and cycleGAN achieved comparable quantitative image similarity metrics, the visual fidelity and robustness to challenging CBCT conditions such as limited FOV and artifacts were notably poorer for the cycleGAN, limiting its reliability for clinical use. Unlike GANs where synthetic images were generated in single steps, diffusion models utilised multi‐steps where only small perturbations were estimated and removed in each step. This suggested that the probabilistic modelling approach of DDPMs could correct errors in later steps, allowing for more faithful reconstruction of CT‐like features from noisy CBCTs.

Unlike Peng et al.’s work,^26^ we were unable to always generate good sCT images with just channel‐conditioning, as seen from the noisy sCT images generated using *t_con_* = 1000. This could be due to our smaller dataset, which had fewer patients and about a third of their slices, or differences in the quality of the pCT and CBCT images used for training and sampling. When noised‐conditioning was used together with channel‐conditioning, the model was able to generate better‐quality sCT images. The examples in Figure 4 demonstrated that the generated sCT patient images successfully reduced metal streaking artifacts from dental implants and preserved the anatomical variations in the CBCT image. These improvements were clearly reflected in the HU difference maps, which showed closer alignment of sCT to pCT compared to the original CBCT. Furthermore, the intensity histograms showed that the distribution of CT numbers in the sCT images more closely resembled those of the pCT than the CBCT, suggesting higher fidelity. Taken together, these findings indicate that the sCT images not only retained the updated anatomical geometry captured in the daily CBCT scans, but also restored HU values to a level more consistent with fan‐beam pCTs. As a result, sCTs might offer a more reliable basis for downstream tasks such as daily segmentation and dose evaluation, potentially outperforming both CBCT or pCT for adaptive radiotherapy workflows. From Figure S‐2c, the phantom pCT images had the same uniform HU value for tissue, while the sCT images generated non‐uniform HU values in the tissue regions. This was likely because the model had learned to generate non‐uniform HU values from real patient examples, and the noisy streaks in the CBCT images had caused the model to generate non‐uniform HU values. Nonetheless, the phantom examples demonstrated that the histograms of CT numbers for generated sCT images followed closely to those of pCT images. In addition, the set‐up differences of the phantom headrest between the pCT and CBCT were not observed in the sCT images, highlighting the utility of sCT images for accurate daily dose evaluation.

From Table 2, the evaluation metrics on the external SynthRAD dataset, using the weights trained solely on the CUH dataset, showed improvements in sCT quality over CBCT in terms of MAE and NCC but yielded a lower PSNR. This was expected, as the model had only been trained on the CUH dataset which differed from SynthRAD in both CT and CBCT acquisition protocols and image characteristics, making the task out‐of‐distribution. These results suggested that while the model exhibited some degree of robustness to unseen data, its ability to generalise across domains remained limited. Importantly, once the model was retrained on the 27 SynthRAD patients for the same duration of time, the evaluation metrics improved significantly across the board. This underscored the importance of dataset‐specific fine‐tuning as a necessary step when adapting the model to new clinical domains. Future work could explore federated learning strategies to enhance cross‐domain performance and generalisability without patient data sharing.^38^ From Figure S‐2a, the examples demonstrated the model's ability to adapt to variations in patient setup, preserving relevant anatomical features from the CBCT even when they differed from the pCT. In particular, the reproduction of the treatment bolus in the sCT underscored the model's potential for capturing daily treatment conditions, which was essential for accurate dose evaluation in adaptive radiotherapy. However, the limited artifact reduction observed in the third case suggested that further work was needed to enhance robustness in challenging scenarios with severe image artifacts. Overall, these findings highlighted the benefit of using sCTs over pCTs for daily dose assessment in image‐guided radiotherapy.

A notable challenge in sCT generation for the pelvic region was the accurate reproduction of bowel gas pockets, which were highly time‐dependent and varied due to peristalsis, bowel loading, and differences in patient preparation. As a result, even rigidly registered pCT‐CBCT pairs often showed mismatched internal anatomy, complicating training. This was particularly problematic for diffusion‐based generative models, which reconstructed images gradually from noise and relied on consistent supervision. In our evaluation, while some bowel gas pockets were accurately synthesised, others were missed, reflecting this limitation. Future work could address this by using pCT and CBCT images acquired on the same day to further minimise inter‐fraction anatomical differences. Despite the limitations, the generated sCT images closely resembled the pCT in terms of image quality and HU distribution and significantly outperformed CBCT in all evaluation metrics. These results supported the feasibility of using our method for pelvis sCT generation in clinically relevant scenarios.

As illustrated in Figure 1, noised‐conditioning worked on the assumption that when added sufficient noise, the structurally similar inputs (i.e., “diffused” pCT and CBCT representations) converged in the latent space into Gaussian noise with overlapping distributions. Figure 2c showed that the cosine similarity and Euclidean distance (which described the similarity and distance between two vectors in a vector space) were approaching 1 and 0 respectively as more noise were added to the pCT and CBCT images. As such, by adding noise to the guiding CBCT image, the model was able to de‐noise from the noisy CBCT image and generate a CT‐quality sCT image. However, different noise schedules degraded the data at different rates. As seen from Figure 2b, the cosine noise schedule degraded the data at a slower rate and retained more information at the beginning of the forward diffusion process, compared to the linear and sigmoid schedules. This was also observed in Figure 2a, where the structure of the body was still visible at the 900th timestep of the cosine schedule, while this information was already lost after the 600^th^ timestep of the linear and sigmoid schedules. This could explain why the sCT evaluation metrics in Table 1 improved most for cosine schedule from *t_con_* = 1000 to *t_con_* = 900, while the improvement for the linear and sigmoid schedules were more gradual and the metrics only achieved cosine‐level quality at *t_con_* = 600. An added advantage of the noised‐conditioning mechanism was the time savings from generating the image. As the sampling process was performed at every timestep, using fewer timesteps would result in faster sampling, which was advantageous in online adaptive workflows where the patient still lay on the treatment couch.

While Figure S‐1 showed that using exponential or triangular timestep distributions would result in a faster loss convergence for the lower timestep bands compared to a uniform distribution, the sCT evaluation metrics did not show improvements over the uniform distribution. This was likely because the differences in loss convergence rate were only significant at the beginning epochs, and the effect of the choice of timestep distributions was diminished when trained over more epochs. Nonetheless, the choice of timestep distributions could matter to clinics with limited computational resources and could only afford to train on fewer epochs.

The findings of our sensitivity analysis suggested that our proposed approach was robust, even when trained on very limited data. Despite a noticeable reduction in training scans, the model maintained high performance across key evaluation metrics, with only marginal degradation. The ability to preserve anatomical structure and achieve clinically relevant image quality with as few as five patient scans highlighted the efficiency of our method, making it particularly suitable for data‐scarce clinical settings or rare disease cohorts. However, signs of overfitting with smaller training sets warranted caution, underscoring the need for appropriate regularisation and monitoring strategies in such low‐data regimes.

From the clinical perspective, it was critical for the sCT images to have superior image quality over the CBCT images to facilitate CBCT‐based segmentation and dose re‐calculations. As such, the reduction in metal artefacts (which, in some cases, were present in both the CBCT and pCT images but not the sCT image) and the preservation of anatomical changes from the CBCT images (e.g., changes in body contour after the pCT was acquired) provided the greatest utility of our sCT images, as dose evaluation using the pCT images would not be as accurate. This was supported by the 3 clinicians’ feedback, where they preferred the sCT over pCT for segmentation and dose evaluation because of reduced artefacts and better mimicking of changes in body contours. In some cases where clinicians preferred the pCTs, it was because the blood vessels looked clearer than the sCTs. In other cases where there were no substantial differences in the two scans, pCTs were also preferred for safety. While the model occasionally generated set‐up BB ball and mask hallucinations, these items would be excluded from the body contour by operators and hence would not affect dose re‐calculations. Further, this issue could be mitigated by pre‐processing the pCT scans to mask out any fiducial markers before using them in training, thereby reducing the likelihood of the model learning such artefacts.

The model was observed to perform worse when the guiding CBCT image was of poor quality, or if the images were acquired from a different centre. This was expected as the model only used information from the guiding CBCT but not the pCT image, hence the generated sCT images were limited by the quality of the CBCT images. Future work could explore the use of both the CBCT and pCT images for guided sampling.

The major limitation of this study was the lack of perfectly registered CT‐CBCT image pairs for both training and testing. By using pCT and CBCT images which were acquired days or weeks apart, there could be residual mismatches due to anatomical changes (e.g., weight loss, tumour growth or shrinkage) or set‐up errors between the images, leading to imperfect correspondence. The model could unintentionally learn these differences and generate inaccurate sCT images. Several recent studies had explored unsupervised diffusion models for CBCT‐to‐CT synthesis to address the challenge of unavailable voxel‐aligned CBCT‐pCT pairs. Fu et al. proposed an energy‐guided diffusion model that introduced handcrafted priors into the sampling process,^39^ while Peng et al. employed a patient‐specific Bayesian score‐based diffusion framework.^40^ These approaches were fully unsupervised, often requiring large training datasets and complex priors to perform well across diverse anatomies. In our approach, we used rigidly registered CBCT‐pCT volumes to extract slice‐level corresponding pairs for training, without requiring deformable registration or voxel‐perfect alignment. While this setup is not entirely unpaired, it reflected a clinically practical weakly supervised regime, where basic anatomical correspondence was sufficient and easy to obtain in real‐world workflows. Importantly, our method demonstrated superior data efficiency, achieving high‐quality CBCT‐to‐CT synthesis using as few as 25 training patients, which was a realistic number for many clinical institutions looking to develop site‐specific models without the need for complex handcrafted priors. This set our approach apart from existing diffusion frameworks, many of which assumed access to extensive datasets and might be impractical to deploy in settings with limited data availability.

Although GAN‐based methods such as cycleGANs had been widely adopted to address the unpaired data limitation,^18^, ^41^, ^42^ they often suffered from hallucination artifacts and structural inconsistencies due to the lack of anatomical constraints. Furthermore, their reliance on adversarial loss could lead to unstable training, especially with small datasets. In contrast, diffusion models offered more stable learning dynamics, and in our case, the integration of channel and noised‐conditioning further ensured anatomical consistency without requiring large datasets. Taken together, our method provided a clinically feasible and anatomically robust solution, balancing the benefits of generative diffusion modelling with the reality of limited training data in radiotherapy departments.

## 5 CONCLUSIONS

This work presented a novel and practical methodology for clinics to train an in‐house DDPM for CBCT‐based sCT image generation using small patient datasets and relatively simple pre‐processing steps. The findings in this work highlighted the novelty and benefit of using our proposed channel and noised‐conditioned sampling method, which was shown to generate high‐quality sCTs with lower MAE and higher PSNR and NCC than GAN‐based sCTs as well as CBCTs on both local and external datasets. We also demonstrated that the sampling approach remained robust even with very small training datasets, though caution was needed to avoid overfitting in low‐data settings.

By reducing the need to acquire repeat CTs for dose evaluation, the sCTs could be used as an additional option to CBCTs for daily positioning, segmentation, dose evaluation, and plan adaptation, resulting in better monitoring of delivered dose to patients while providing potential time and resource savings for the clinics.^43^, ^44^ Our work to date therefore provided externally‐validated proof‐of‐concept that with channel and noised‐conditioned sampling and an optimal choice of timestep sampler, a DDPM could be trained in an individual clinic to provide an enhanced workflow for CBCT‐based synthetic CT generation and adaptive radiotherapy.

For future work, we aimed to test if this approach was image modality agnostic and applicable for other image‐to‐image translation tasks, and perform dose recalculations on the generated sCTs to quantify their impact on adaptive planning accuracy. Further work to develop an uncertainty estimation function in generated sCT images would provide greater utility in assessing sCT suitability for dose re‐calculations.^45^, ^46^, ^47^, ^48^ We also planned to investigate if the fidelity of sCT images can be improved by fine‐tuning the model on weakly‐supervised pre‐trained networks trained with synthetic CBCT images generated by Monte Carlo simulations. With access to better computational facilities, the use of 3D DDPMs to generate 3D sCT scans could be explored.

## CONFLICT OF INTEREST STATEMENT

The authors have no relevant conflicts of interest to disclose.

## Supporting information



Supporting information

## Data Availability

The data that support the findings of this study are available from Cambridge University Hospitals but restrictions apply to the availability of these data, which were used under licence for the current study, and so are not publicly available. The SynthRAD2023 Grand Challenge dataset was available at: https://github.com/SynthRAD2023.
