# Supplementary material for: Few‐shot CBCT‐based synthetic CT generation with denoising diffusion probabilistic model
Source: Med Phys. 2025 Nov 13;52(11):e70126. doi: 10.1002/mp.70126 (PMC12614291; doi:10.1002/mp.70126)
Supplement: Supplementary file 1 — Supporting information [file MP-52-0-s001.docx]

***Supporting Material***

**Evaluation Metrics**

The evaluation metrics for sCT images are described by the following equations:

$$\begin{aligned} \mathrm{MAE}=\frac{1}{n_{x}n_{y}}\sum_{i,j}^{n_{x},n_{y}} |\mathrm{CT}_{1}(i,j)-\mathrm{CT}_{2}(i,j)| \#\left( 7 \right) \end{aligned}$$

$$\begin{aligned} \mathrm{PSNR}=10\times\log_{10} \left( \frac{{MAX}^{2}}{\frac{1}{n_{x}n_{y}}\sum_{i,j}^{n_{x},n_{y}} {|\mathrm{CT}_{1}(i,j)-\mathrm{CT}_{2}(i,j)|}^{2}} \right) \#\left( 8 \right) \end{aligned}$$

$$\begin{aligned} \mathrm{NCC}=\frac{1}{n_{x}n_{y}}\sum_{i,j}^{n_{x},n_{y}} \frac{(\mathrm{CT}_{1}(i,j)-\bar{\mathrm{CT}_{1}})(\mathrm{CT}_{2}(i,j)-\bar{\mathrm{CT}_{2}})}{\sigma_{\mathrm{CT}_{1}}\sigma_{\mathrm{CT}_{2}}} \#\left( 9 \right) \end{aligned}$$

where $\mathrm{CT}(i,j)$ is the value at pixel $(i,j)$ of the $\mathrm{CT}$ image, $n_{x}$ and $n_{y}$ are the number of pixels along the image dimensions, $MAX$ is the maximum pixel value in the two images, $\bar{\mathrm{CT}}$ is the mean pixel value of the image, and $\sigma_{\mathrm{CT}}$ is the standard deviation of the image.


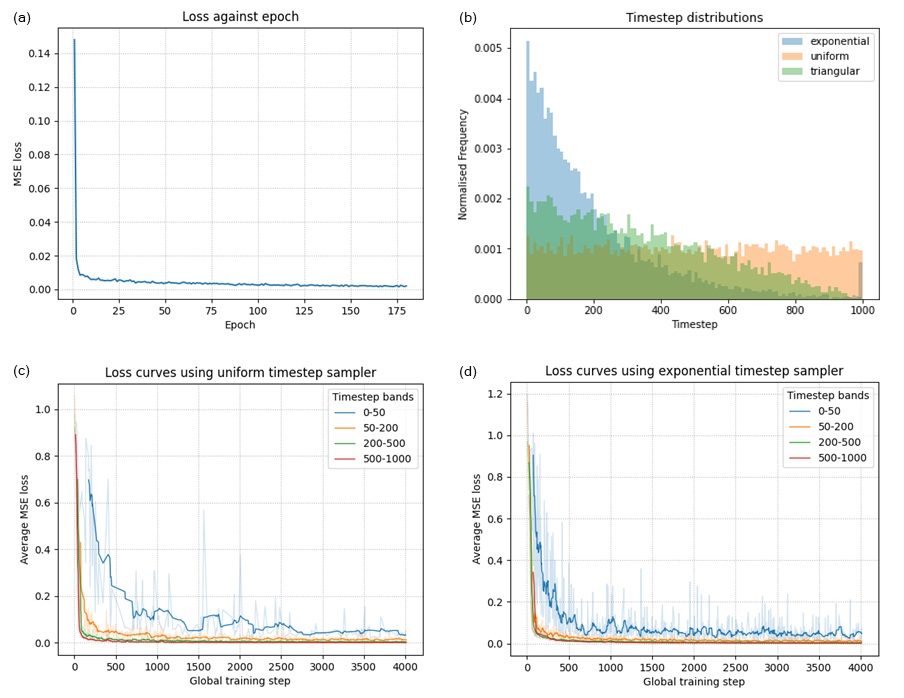


**Figure S-1: Loss convergence with different timestep sampling distributions.** (a) Plot of loss aggregated across all batches over 180 epochs. (b) Histograms of exponential (blue), uniform (orange), and triangular (green) timestep sampling distributions. Loss curves of different timestep bands for (c) uniform and (d) exponential timestep sampling distribution. The lighter plot shows the individual loss values, while the darker plot shows the moving average.


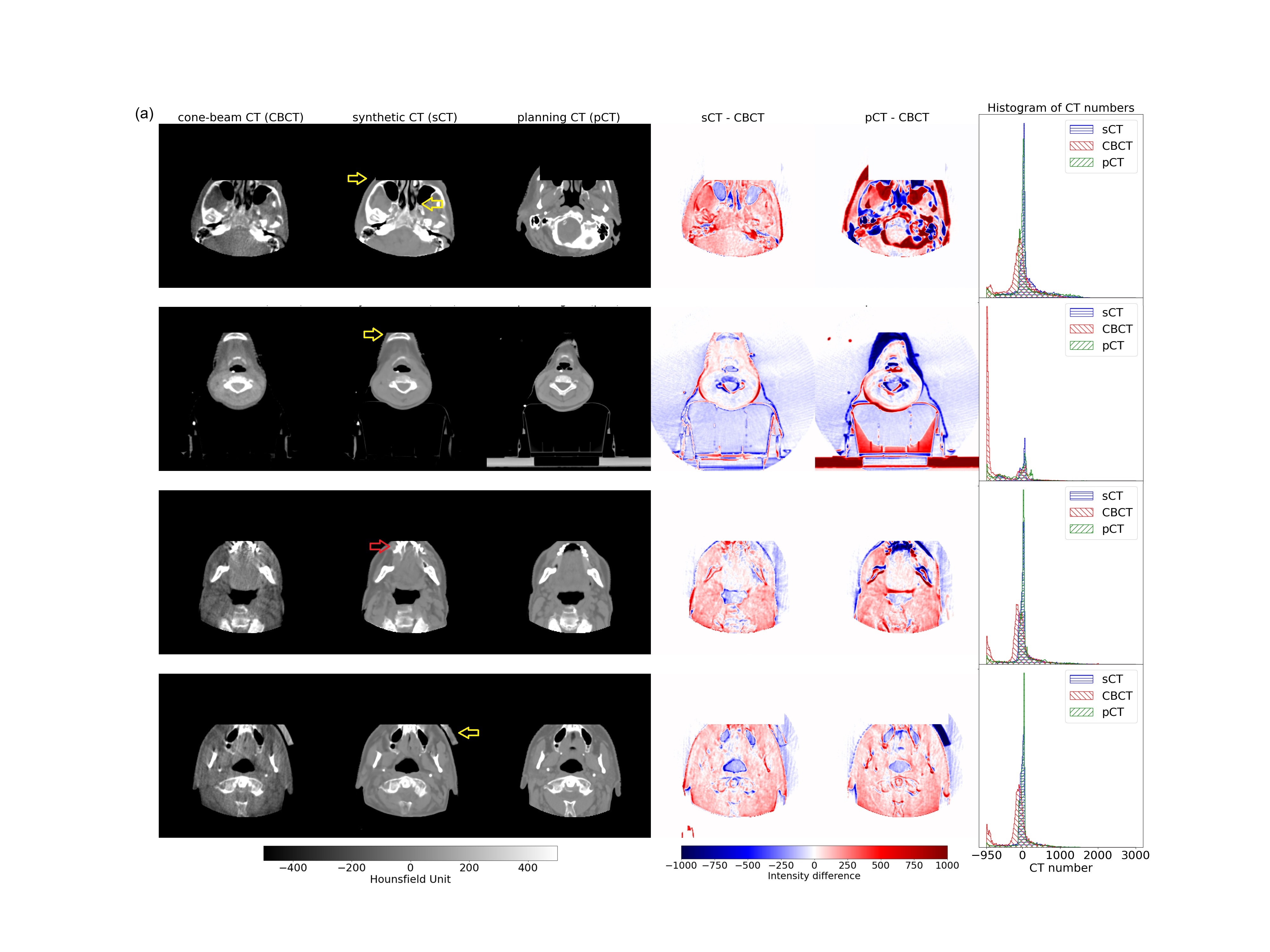

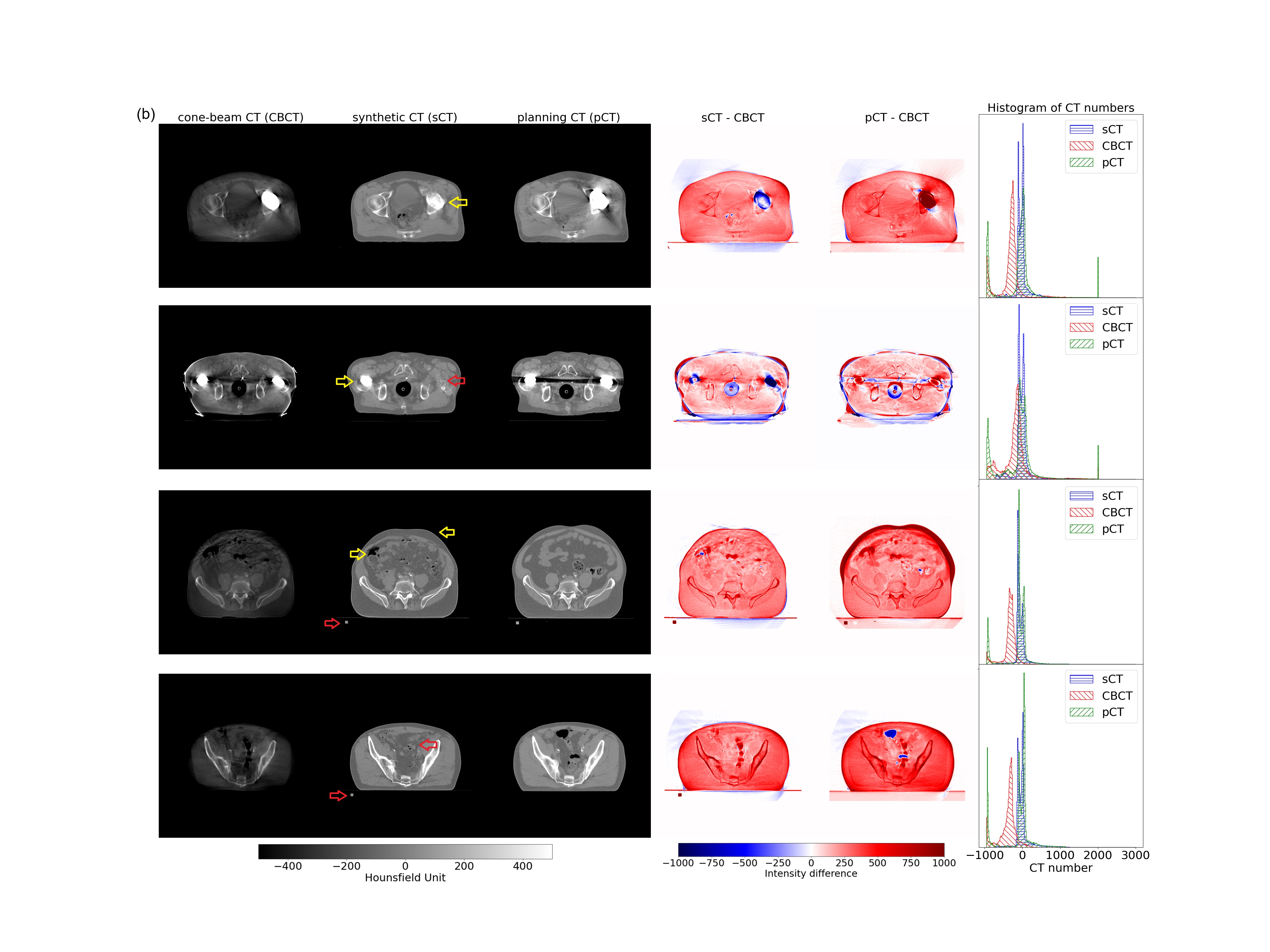

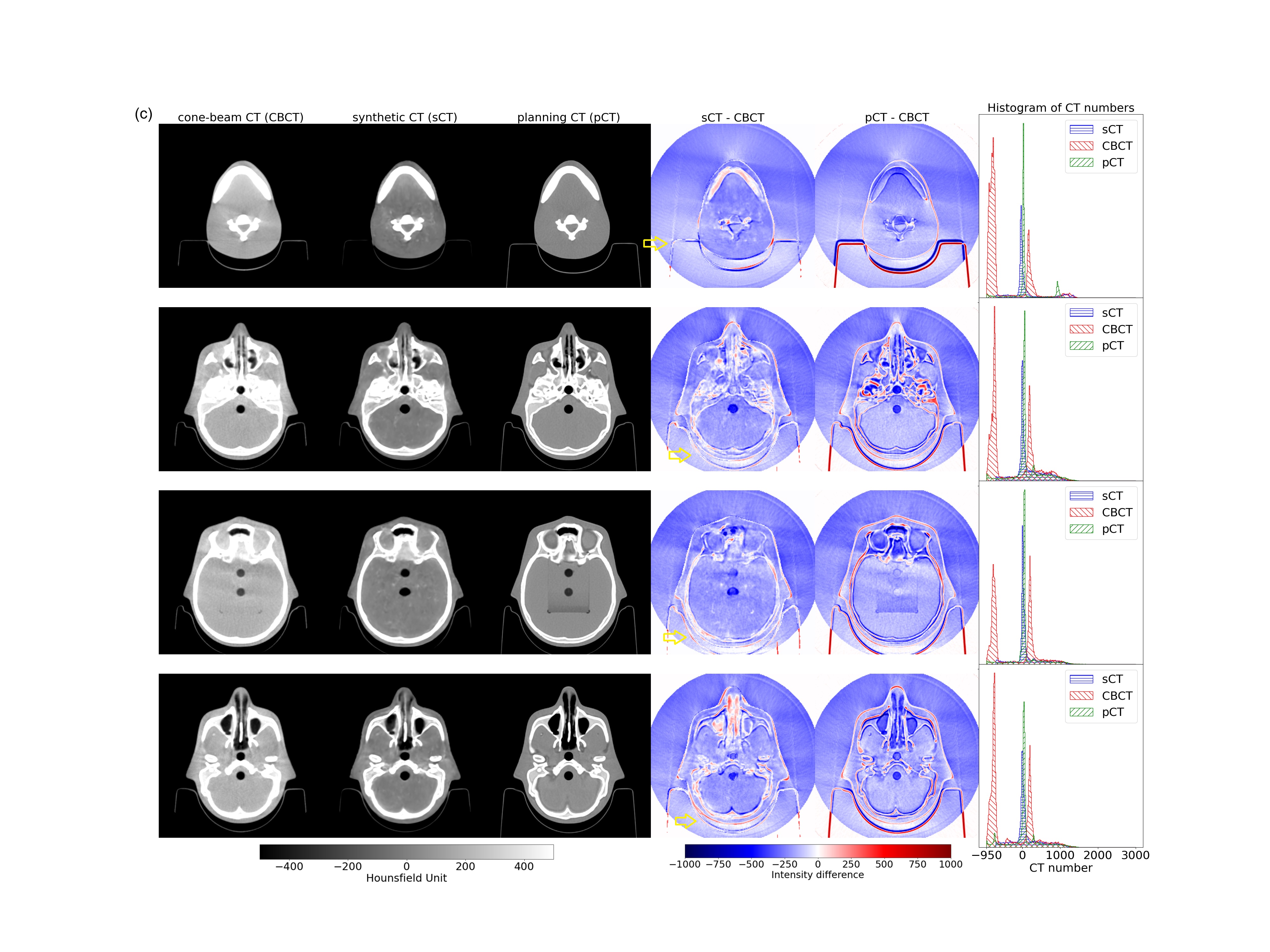


**Figure S-2: Sample images of generated sCT with the guiding CBCT and corresponding pCT images, HU difference maps, and histogram of CT numbers for (a) SynthRAD H&N, (b) SynthRAD pelvis, and (c) phantom H&N.** Improved sCT features were annotated with yellow arrows, while poor-performing sCT features were annotated with red arrows.


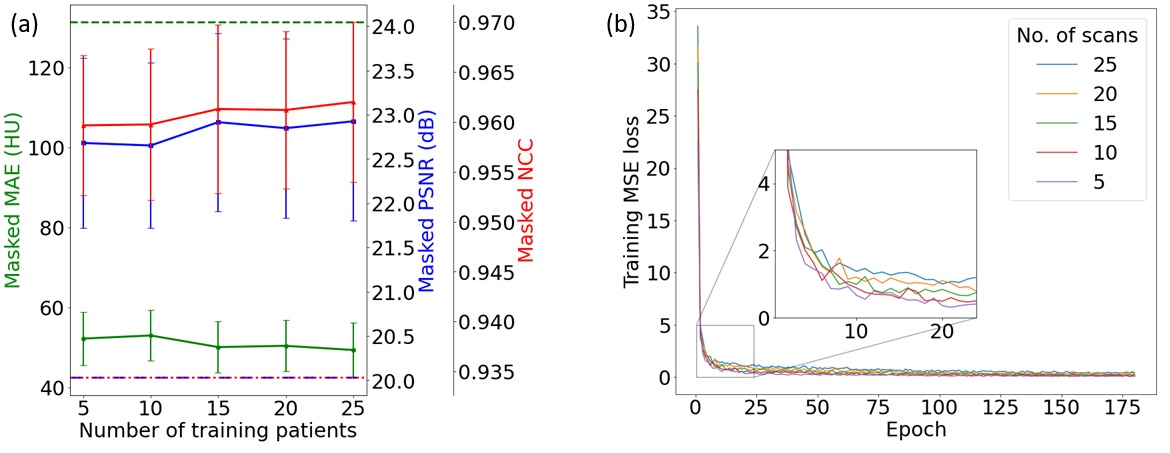

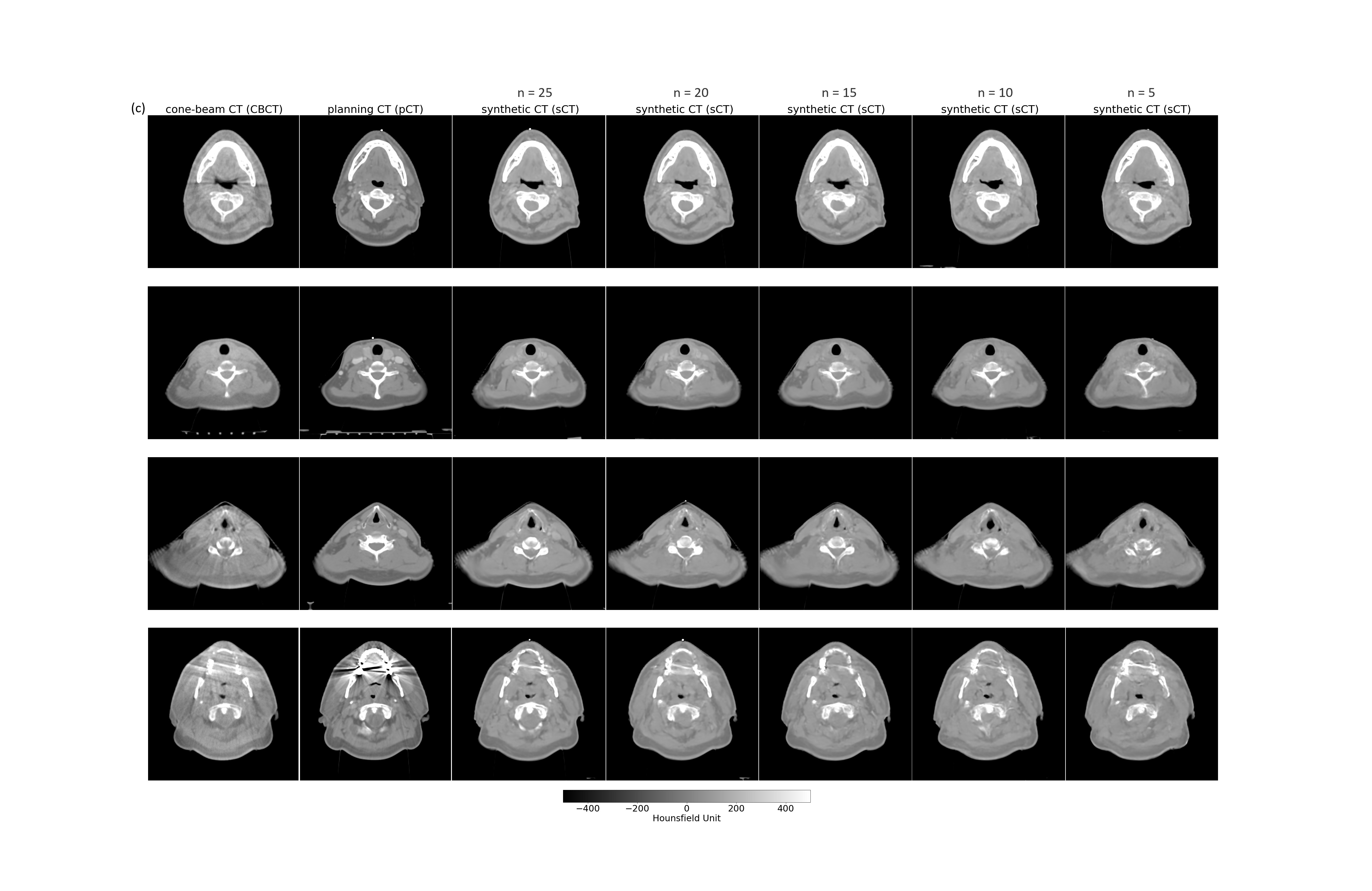


**Figure S-3: Sensitivity analysis with progressively smaller patient training set, showing (a) evaluation metrics, (b) training loss, and (c) example images.** In Fig. 7(a), the green, blue, and red lines give the masked MAE, PSNR, and NCC, respectively. The solid lines show the sCT metrics for different training data size, while the dashed lines show the CBCT metrics.
